# Thermodynamics of primary photosynthesis

**DOI:** 10.1007/s11120-013-9919-x

**Published:** 2013-09-14

**Authors:** D. Mauzerall

**Affiliations:** Rockefeller University, 1230 York Ave, New York, NY 10065 USA

**Keywords:** Black body, Equilibrium, Irreversible process, Kinetics, Temperature

## Abstract

The thermodynamics of photosynthesis has been much discussed, but recent articles have pointed to some confusion on the subject. The aim of this review is to clarify a limited part of this state of affairs.

## Introduction

Early discussions of the thermodynamics of photosynthesis concluded that the efficiency is inherently limited (Duysens 1958; for a good review see Knox 1969). More recently, Lavergne and Joliot (2000) proposed a similar efficiency limit of ~70 % based on the Carnot cycle and a “temperature” of ~1,100 K for the excited state of chlorophyll. However, Parson (1978) had already argued that the Carnot cycle was not applicable and that the kinetics of the species determined the efficiency. Jennings et al. (2005) have reviewed this literature and come down on the side of Parson but with rather distressing conclusions on the violation of the second law of thermodynamics. This has been refuted by Lavergne (2006) and by Knox and Parson (2007). Jennings et al. (2007) disagree but offer no refutation. I believe Lavergne and Knox and Parson are correct, but their arguments are based on implicit assumption of equilibrium between radiation and the excited state. The limited aim of this review is to discuss the efficiency of the primary reactions of photosynthesis. This is critical since the overall yield completely depends on the initial yield.

## Temperature and irreversibility

An important aspect of the matter lies in the hypothetical “radiation temperature” assigned to the light beam. This concept originates in Planck’s view of assigning an entropy, and thus a temperature, to radiation. However, Planck was very clear that there is only one unique thermodynamic radiation temperature: that of the black body at equilibrium (Planck 1912). In fact, he states that since rays of radiation, used to define a temperature, passing through a point can be arbitrary, there are an infinite number of such “temperatures”. Almost all of the previous discussions have used these arbitrary “temperatures” in thermodynamic equations that require equilibrium to be exact.

A simple view of the situation is to say that once the photon is absorbed and the excited state formed, it has no memory whatsoever of the source of the photon: this is an irreversible process in complex molecules. Once one knows the quantum yield of the process and the free energy of the products, it is a straightforward matter to calculate the fraction of solar energy converted to stored energy: it is the ratio of the energy of the products divided by the integrated absorption of the solar energy. Note that the technique of photoacoustics allows just this fraction to be precisely determined (Mielke et al. 2011). The quantum yield may be almost 100 % as it is in the primary reaction of photosynthesis. This yield is determined by kinetics: the ratio of the rate to products divided by the sum of this and of all competing processes. A high yield is usually associated with a loss of free energy in forming products via intermediates. This loss provides a thermal barrier to the equilibration of the intermediates with the excited state and thus minimizes loss of the excitation energy and increase of efficiency of energy storage.

The discussion will be restricted to the efficiency of the primary reaction of energy storage. Given this simple view, the only relevant parameter is the energy of the absorbed photons, as Parson (1978) has indicated. To be thermodynamically specific, this energy is an enthalpy since the energy of the light beam is always ultimately measured as the heat liberated on total absorption and decay. This also follows from the simple view of loss of memory on absorption. Quantum meters are generally unavailable since all detectors have unknown absolute sensitivity, which usually varies with wavelength. Thus the number flux of photons in the light beam is simply the energy flux divided by Planck’s constant times the frequency, with a suitable average over the distribution of frequencies if required. The much-used notion of the temperature of a photon flux is valid only for the black body distribution of frequencies, since this is an equilibrium situation with a well-defined temperature, the thermodynamic temperature. All other “temperatures” depend on definitions. In any case, they are irrelevant as the simple view states. Essentially, the absorption of a photon—at the intensities and for molecules relevant to photosynthesis—is an irreversible process, and its description as an equilibrium process leads to the aforementioned confusion.

## Free energy and equilibrium

The free energy of a process can only be defined for the process at equilibrium. Measuring the free energy via the redox potentials of short-lived excited states is difficult, requiring electron transfer equilibrium to be obtained during the lifetime of the state. For simple molecules in a non-reactive environment, the energy of the equilibrated excited state is usually taken to be the crossing point of the normalized absorption and fluorescence spectra. This is required because of the Stokes shift in polyatomic molecules. This shift measures the difference of the vibe-rot-librational frequencies, including interactions with the solvent, between the ground and excited states of the molecule and their differing interactions with the surrounding medium. It can be small (e.g., ~0.03 eV for chlorophyll) or large (e.g., ~1 eV for some aromatic amines used as polarity “reporter” groups). [Note that one way to obtain an efficiency >100 % is to excite the molecule at a frequency less than the maximum of the fluorescence emission band. In this case, thermal energy is used to re-equilibrate the excited state. This is the method used to prepare ultra cold gas atoms (Bose condensates) and has even been observed in the liquid phase with rhodamine 6G (Zander and Drexhage 1995). Our recent measurements on the chlorophyll *d* containing *A. marina* (Mielke et al. 2013) show that the slowing of the forward reaction by the necessary uphill activation energy actually decreases the efficiency of energy storage.]

The assumption that the energy of the thermally equilibrated excited state is the free energy is reasonable if the entropy change is small, as is the case in chlorophyll. An efficiency of >98 % for the primary reaction of green plant photosynthesis when excited at the main absorption band is thus allowed. The energy of the first cation–anion pair in photosynthesis is not precisely known but the efficiency is ~95 %. Since the first step in photosynthesis is electron transfer, its yield depends on the rate of reverse electron transfer, assuming the other deactivation paths are slow as is required for maximum efficiency. As has been pointed out repeatedly, for a yield of *X* % one needs a reverse rate of (100 − *X*) % of the forward rate. This is usually written as the energy loss in the forward step to enable the minimum thermodynamically required slowing of the reverse step via a Boltzmann distribution. However, as I have pointed out, there is more than one way to skin a cat: at least a half-dozen, and these are unlikely to have exhausted the subject (Mauzerall 1988). Quantum mechanics in particular allows a variety of possibilities.

The simple Boltzmann-based argument of slowing the reverse rate leads to the requirement of 0.6 eV decrease of free energy to ensure a 99 % yield of product on the 1 ms time scale required to form oxygen, given a forward reaction time of 3 ps. On the 10 s timescale of the most stable S-state of the oxygen forming cycle, which allows photosynthesis in very dim light, the required energy loss is 0.83 eV. Thus, a thermal efficiency of 54 % from a 680 nm (1.8 eV) photon is possible. The measured efficiency at the trap energy is ~35 % (Mielke et al. 2011) so some gain is theoretically possible. However, this efficiency is very close to that delivered by the final products of photosynthesis, oxygen and glucose, if eight photons are required for the complete cycle. It may be difficult to outdo evolution.

Exactly because it is a photochemical system, the thermal efficiency of photosynthesis is wavelength dependent: it decreases with decreasing wavelength. The energy of all photons greater than the equilibrated energy of the excited state is immediately degraded to heat. This is another reason why the thermal or Carnot cycle arguments are irrelevant. The efficiency then depends on the assumed “temperature” of the light source, which increases with decreasing wavelength. In fact the thermal efficiency depends in large part on the choice of the trap energy—i.e., the energy of the primary reaction—by evolution. This is clearly seen in the classic paper on the efficiency of photovoltaic devices by Shockley and Queisser (1961). They use only one temperature in their arguments, that of the sun, but stress the role of the energy gap in determining the efficiency of the device. The energy gap is equivalent to the trap energy in photosynthesis and clearly has nothing to do with the light source.

In the beginning of the evolution of photosynthesis, the trap energy was determined by available molecular absorbers, donors and acceptors. Nowadays, it is determined by the requirement to use water as the source of reducing equivalents. This requirement has focused interest on the minimal trap energy required for the production of its complement, oxygen. The methodology of photoacoustics allows the direct measurement of trap energies (Mielke et al. 2013). Our measurements on *A. marina*, which uses chlorophyll *d* absorbing some 40 nm to the red of chlorophyll *a*, indicate a similar efficiency of the photosystems (Mielke et al. 2011). Thus, the reduction of excitation energy in the case of *A. marina* has not reached the minimum energy required for using water as the primary donor.

The complication of predicting this trap energy in photosynthesis is the Jekyll–Hyde effect of the protein. On the one hand, holding the redox molecules at the optimum distance and orientation to provide the ideal environment are what produce the observed unity quantum yields of charge separation via quantum mechanical tunneling of electrons. On the other hand, the innate flexibility of proteins, and their ungodly number of degrees of freedom, almost ensure that the thermal relaxations will extend over a wide range of time scales. All measurements seem to converge on this last point (see, e.g., Parson 1982; Woodbury and Allen 1995; Xu and Gunner 2000; de Winter and Boxer 2003). The result is that the system is not at thermal equilibrium during some stages of the reaction. Its free energy is therefore not well-defined, and it can only be described by methods of irreversible thermodynamics. Note that the enthalpy and entropy changes are still meaningful; in fact, the excess entropy change, i.e., an entropy more positive than the equilibrium value, can be used as the criterion of irreversibility.

## Summary

Considerations of thermal machines are irrelevant to the efficiency of photosynthetic reactions since these are essentially isothermal photochemical processes. The efficiency of converting the energy of the absorbed photon to free energy of products is limited only by kinetics: the ratio of loss channels to the product channel as stated by Parson (1978). If the losses were negligible, the efficiency could be >98 %. With a realistic estimate of the kinetically required loss reactions, the efficiency from the trap energy could be 54 %. The efficiency of forming oxygen and glucose from water and carbon dioxide, assuming eight photons at 680 nm are required, is close to the observed efficiency, 35 %, so it may be difficult to improve on evolution.

Because the photosynthetic system is “open”, one can explain the observed effects of energy storage, including the vexing order-producing or “emergent” properties, without recourse to violations of the Second Law of Thermodynamics as claimed by Jennings et al. (2005).
